# Supplementary material for: Complexome profiling of the Chlamydomonas psb28 mutant reveals TEF5 as an early PSII assembly factor
Source: Plant Cell. 2025 Mar 17;37(6):koaf055. doi: 10.1093/plcell/koaf055 (PMC12164590; doi:10.1093/plcell/koaf055)
Supplement: koaf055_Supplementary_Data [file koaf055_supplementary_data.zip › psb28_interactive.zip!/plots/intensity/qualityPlots/peptides/MOT20_Cre09.g415900.t1.2.html]

### Cre09.g415900.t1.2

Trivial name: MOT20  
SeqCoverage [median]: 4.80  
Mass: 36.31 kDa  
  
 

| Sequence | MissedCleavage | Mass [Da] | wtIntensity | muIntensity | Score |
| --- | --- | --- | --- | --- | --- |
| SDDLEKQPLVQAGGKN | 2 | 1697.86 | 0.00 | 817.60 | 76.76 |
|  |  |  | 0.0 | 817.6 |  |
